# Supplementary material for: Incidence and risk factors associated with acquired syphilis in HIV pre-exposure prophylaxis users
Source: PLoS One. 2024 Jul 5;19(7):e0303320. doi: 10.1371/journal.pone.0303320 (PMC11226132; doi:10.1371/journal.pone.0303320)
Supplement: S1 File — (DOCX) [file pone.0303320.s001.docx]

**Supporting Information**

**S1 Support information. Forms to assist PrEP users, Brazil**

These forms were used at the time of data collection are available at the links below (in Portuguese):

• Registration form: <http://azt.aids.gov.br/documentos/prep_resumida_ficha_cadastro_paciente_digitavel.pdf> ;

• First visit form: <http://azt.aids.gov.br/documentos/02_Ficha_Primeiro%20Atendimento_PrEP.pdf> ;

• Monitoring form: <http://azt.aids.gov.br/documentos/04_Ficha_Monitoramento_PrEP.pdf> .
