# Supplementary material for: Incidence and risk factors associated with acquired syphilis in HIV pre-exposure prophylaxis users
Source: PLoS One. 2024 Jul 5;19(7):e0303320. doi: 10.1371/journal.pone.0303320 (PMC11226132; doi:10.1371/journal.pone.0303320)
Supplement: S2 File — (DOCX) [file pone.0303320.s002.docx]

**Supporting Information**

**S2 Support information. Qualification of the research database**

The process of qualifying the database of users of Pre-exposure Prophylaxis (PrEP) for HIV was conducted throughout Brazil, from 2018 to 2020, using the R® 4.3.3 software.

The databases with the data mentioned above were facilitated by the Ministry of Health and contain registration information, PrEP behavioral criteria, and other health data. Database 1 (BD1) contains user registration data from the study period, with data individualized by “id”.

The variables used in this database were “id”, sex at birth, gender identity, self-reported ethnicity/skin color, and schooling, totaling 38,397 observations of five variables of interest. Database 2 (BD2) contains behavioral data, complementing the user’s registration data and their PrEP eligibility criteria.

The variables of interest in this database were “*I have already been diagnosed with syphilis*” and “*PrEP patient code*”, with a total of 35,915 records for 2 variables. Bank 3 (BD3) contains data on cases of active syphilis on the user’s return 30 days after the first PrEP dispensation, with 27,993 records. Unlike the other databases, Database 4 (BD4) has data for each monitoring visit; each observation corresponds to a follow-up visit, identified by the “*PrEP patient code*”, with 79,761 records, and its 18 variables of interest were number of sexual partners in the last 3 months (split by men, women, trans men, trans women, and transvestites), frequency of condom use in the last three months, alcohol abuse, recreational substance use (segregated by poppers, cocaine, crack, marijuana, club drugs, erection stimulants, and solvents), injecting drug use, and completion of an active syphilis test and age.

The linkage procedures were conducted in stages to build the work’s database. Initially, we investigated and deduplicated the databases using a deterministic approach based on the variable “id” (or “*PreP patient code*”). DB1 had no deduplicated lines, keeping its 38,397 records. DB2 had 1,915 duplicate lines (5.3%), keeping 34,000 records. DB3 had 802 duplicate records (2.8%), keeping 27,191 records. DB4 underwent a different process to extract the observations of interest because it contained user queries. After this stage, we performed an inner join of BD1 and BD2 (BD12), totaling 34,000 records. There were no losses for BD2, with losses of 4,397 records (11.5%) for BD1.

Before extracting a new database with the appointments of interest for our analysis, we identified the total number of new cases of active syphilis identified in the monitoring visits and integrated them into our database. We extracted the most recent appointments of each PrEP user from BD4 as a parameter for the behavioral variables used in our analysis.

We found that 20,203 users had made at least one monitoring appointment in the period. By joining the database with the data from the first appointment with the database from the last monitoring appointment using the left join process, we obtained a database of 19,820 people monitored for PrEP (bdfinal).

The figure below shows the relationship between the databases. Finally, the following packages and scripts were used in the software:

pacman::p_load(

tidyverse,

janitor,

stringr,

rio,

here,

purrr,

gtsummary,

broom,

lmtest,

parameters,

see,

readxl,

plyr)

BD1 <- read_excel("C:\\00 NATHALIA DOC\\BD1.xlsx")

View(BD1)

BD2 <- read_excel("C:\\00 NATHALIA DOC\\BD2.xlsx")

View(BD2)

BD3 <- read_excel("C:\\00 NATHALIA DOC\\BD4.xlsx")

View(BD3)

BD4 <- read_excel("C:\\00 NATHALIA DOC\\BD4.xlsx")

View(BD4)

BD1_unique %>%

janitor::get_dupes(`id`)

BD2_unique <- distinct(BD2,`00 Codigo Paciente Prep`,.keep_all = TRUE)

BD2_unique %>%

janitor::get_dupes(`00 Codigo Paciente Prep`)

BD3_unique <- distinct(BD2,`00 Codigo Paciente Prep`,.keep_all = TRUE)

BD3_unique %>%

janitor::get_dupes(`00 Codigo Paciente Prep`)

BD2_unique <- rename(BD2_unique, id =`00 Codigo Paciente Prep`)

BD4 <- rename(BD4, id =`00 Codigo Paciente Prep`)

BD3_unique <- rename(BD3_unique, id =`00 Codigo Paciente Prep`)

BD1 <- transform(BD1, id=as.character(id))

BD2_unique <- transform(BD2_unique, id=as.character(id))

bd12 <- inner_join(BD1,BD2_unique, by = "id")

BD4sifilis <- filter(BD4,`21 Resultado teste sifilis ativa`== "Sim" | `21 Resultado teste sifilis ativa`== "sim")

BD4qtdesifilis <- BD4sifilis %>% group_by(id) %>% count()

bd12sif <- left_join(bd12, BD4qtdesifilis, by = "id")

bd12 <- transform(bd12, id=as.character(id))

BD4qtdesifilis <- transform(BD4qtdesifilis, id=as.character(id))

bd12sif <- left_join(bd12, BD4qtdesifilis, by = "id")

bd12sif <- rename(bd12sif, n_infeccoes_sifilis = n)

bd12sif <- bd12sif %>% mutate(n_infeccoes_sifilis= ifelse(is.na(n_infeccoes_sifilis),0,n_infeccoes_sifilis))

bd4_id_unique <- unique(BD4["id"])

bd4_final <- data.frame()

BDTemp <- data.frame()

x <- as.list(bd4_id_unique["id"])

x

for(i in 1:length(bd4_id_unique$id)) {

BDTemp <- filter(BD4, id == bd4_id_unique$id[i])

BDTemp <- arrange(BDTemp, desc(`03 Data preenchimento`))

linha <- head(BDTemp, 1)

bd4_final <- rbind(bd4_final, linha)}

bdfinal <- inner_join(bd12sif,bd4_final, by = "id")
