# Supplementary material for: Incidence and risk factors associated with acquired syphilis in HIV pre-exposure prophylaxis users
Source: PLoS One. 2024 Jul 5;19(7):e0303320. doi: 10.1371/journal.pone.0303320 (PMC11226132; doi:10.1371/journal.pone.0303320)
Supplement: S1 Table — (DOCX) [file pone.0303320.s004.docx]

**Supporting Information**

**S1 Table. Variance inflation factor (VIF) of the multivariate model of factors associated with syphilis during HIV Pre-exposure Prophylaxis, Brazil, 2018-2020.**

| **Variable** | VIF | Df |
| --- | --- | --- |
| Age | 1.089735 | 1 |
| Sex at birth | 1.409801 | 2 |
| Sex orientation | 1.531604 | 3 |
| Self-reported ethnicity/skin color | 1.041293 | 4 |
| Accepted something in exchange for sex | 1.177120 | 1 |
| Syphilis history | 1.008737 | 1 |
| Condom frequency | 1.080609 | 4 |
| Number of sexual partners men | 1.146090 | 1 |
| Number of sexual partners women | 1.038854 | 1 |
| Number of sexual partners trans women | 1.005675 | 1 |
| Drug use-Poppers | 1.152290 | 1 |
| Drug use-Cocaine | 1.197142 | 1 |
| Drug use- Marijuana | 1.132814 | 1 |
| Drug use-Erection stimulants | 1.147526 | 1 |

*Df: Degrees of freedom
